# Publisher Correction: npj Vaccines, volume 4

**DOI:** 10.1038/s41541-019-0127-3

**Published:** 2019-08-13

**Authors:** npj Vaccines

**Affiliations:** London, United Kingdom

An error occurred during the publication of a number of articles in *npj Vaccines*. Several articles were published in volume 4 with a duplicate citation number.

In this correction article the old and new citation metadata are published in Table 1.

**Table 1:** Overview of incorrect and correct citation metadata

DOI	Published online date	Incorrect citation number	Correct citation number
10.1038/s41541-019-0117-5	03 June 2019	1	25
10.1038/s41541-019-0118-4	03 June 2019	2	26
10.1038/s41541-019-0119-3	25 June 2019	3	27

The original articles have been updated. The publisher apologises for the inconvenience caused to our authors and readers.

